# Molecular Imaging in Multiple Myeloma—Novel PET Radiotracers Improve Patient Management and Guide Therapy

**DOI:** 10.3389/fnume.2022.801792

**Published:** 2022-02-25

**Authors:** Johannes von Hinten, Malte Kircher, Alexander Dierks, Christian H. Pfob, Takahiro Higuchi, Martin G. Pomper, Steven P. Rowe, Andreas K. Buck, Samuel Samnick, Rudolf A. Werner, Constantin Lapa

**Affiliations:** ^1^Nuclear Medicine, Faculty of Medicine, University of Augsburg, Augsburg, Germany; ^2^Department of Nuclear Medicine, University Hospital Würzburg, Würzburg, Germany; ^3^Graduate School of Medicine, Dentistry and Pharmaceutical Sciences, Okayama University, Okayama, Japan; ^4^The Russell H. Morgan Department of Radiology and Radiological Science, Johns Hopkins University School of Medicine, Baltimore, MD, United States

**Keywords:** [18F]fluorodeoxyglucose, [C11]methionine, [C11]choline, [68Ga]Pentixafor, [177Lu]Penthixather, CXCR4, theranostics

## Abstract

Due to its proven value in imaging of multiple myeloma (MM), including staging, prognostication, and assessment of therapy response, 2-deoxy-2-[^18^F]fluoro-D-glucose (FDG) positron emission tomography (PET) is utilized extensively in the clinic. However, its accuracy is hampered by imperfect sensitivity (e.g., so-called FDG-negative MM) as well as specificity (e.g., inflammatory processes), with common pitfalls including fractures and degenerative changes. Novel approaches providing a read-out of increased protein or lipid membrane syntheses, such as [^11^C]methionine and [^11^C]choline or the C-X-C motif chemokine receptor 4-targeting radiotracer [^68^Ga]Pentixafor, have already been shown to be suitable adjuncts or alternatives to FDG. In the present focused review, those imaging agents along with their theranostic potential in the context of MM are highlighted.

## Introduction

Multiple myeloma (MM) is defined by neoplastic cell proliferation in the bone marrow and by the overproduction of monoclonal antibodies or paraproteins (1). As an orphan disease, 1% of all cancer patients and 10% of all hematologic malignancies are attributable to MM (2, 3). Diagnosis is established based on the recommendations of the International Myeloma Working Group (IMWG) criteria, which are characterized by bone marrow infiltration, monoclonal proteins in body fluids (e.g., serum or urine), and osseous or renal damage (4). Magnetic resonance imaging (MRI) is the reference for detection for involvement of bone marrow and is recommended by the IMWG if prior screening with whole-body computed tomography (CT) does not provide a sufficient read-out of osteolytic bone lesions (5). For instance, as recently demonstrated in a prospective setting, MRI and positron emission tomography/computed tomography with 2-deoxy-2-[^18^F]fluoro-D-glucose (FDG PET/CT) were comparable for assessing bone lesions (6). Relative to PET, however, MRI identifies more diffuse bone lesions, whereas the tracer-based imaging technique is better suited to assess treatment response (6–8). Thus, as it can assess both bone marrow infiltration and extramedullary disease sites, the IMWG recommends FDG PET/CT, not only for suspected disease, but also in relapse or refractory scenarios (9).

In this regard, FDG is considered a work-horse in MM, in particular as it can identify on-going disease (9). As such, FDG PET/CT can assess therapeutic efficacy and the identification of patients at risk of therapeutic failure, e.g., after novel agent-based induction therapy and subsequent double autotransplantation (10). For instance, Sachpekidis et al. investigated 48 treatment-naïve MM patients which underwent FDG PET in a prospective setting prior to autologous stem cell transplantation. Various quantitative and qualitative PET-based parameters could differentiate between high- vs. low-risk individuals. Patients with pathological scan findings on a visual assessment or increased maximum standardized uptake values had shorter progression-free survival (11).

Despite its usefulness in various clinical scenarios, and availability at virtually every PET center, FDG suffers from various drawbacks. For instance, false negative results may occur in low hexokinase-2 expressing MM, which is observed in up to 11% of MM patients (12). As another pitfall, diffuse skeletal involvement or decreased metabolic activity may reduce sensitivity (13). In addition, accurate scan interpretation can be hampered by ongoing inflammation, changes due to degenerative or inflammatory arthritis, or recent fractures—all sources of false positive uptake that potentially compromise the specificity of FDG (14). Other confounding factors include tumor heterogeneity and shifting pathophysiological pathways in advanced disease. In addition, the complexity of an FDG scan in MM patients may lead to inconsistent interpretation of imaging findings. To address this, Nanni et al. evaluated the Italian Myeloma criteria for PET USe (IMPeTUs) criteria (15). In brief, these are based on a visual assessment using the Deauville score and also include morphological findings of radiotracer distribution, e.g., non-focal uptake in the bone marrow or extra-medullary lesions (16). IMPeTUs was reproducible for scans at baseline and during follow-up (15).

As such, the armamentarium of PET radiotracers for MM patients has been expanded to alternatives that are not dependent upon high glycolytic metabolism in the tumor cells. Options include, but are not limited to, [^11^C]/[^18^F]choline (CH), [^11^C]methionine (MET), [^68^Ga]Pentixafor (PEN), [^18^F]-3′-fluoro-3′-deoxythymidine (FLT) and [^18^F]-Sodium Fluoride (NaF) (17–23). In this focused review, novel imaging agents along with their theranostic potential in the context of MM will be highlighted.

## Novel Radiotracers

### [^68^Ga]Pentixafor (PEN)

The C-X-C motif chemokine receptor 4 (CXCR4) can be found in more than 30 different hematopoietic and solid cancers (24–27). In patients with MM, it is involved in homing and, cell mobilization from the bone marrow, potentially leading to distant organ involvement (28). In MM, about half of patients demonstrated distinct CXCR4 overexpression (29, 30), thereby rendering this receptor suitable for both imaging and therapy.

**Figure 1:**
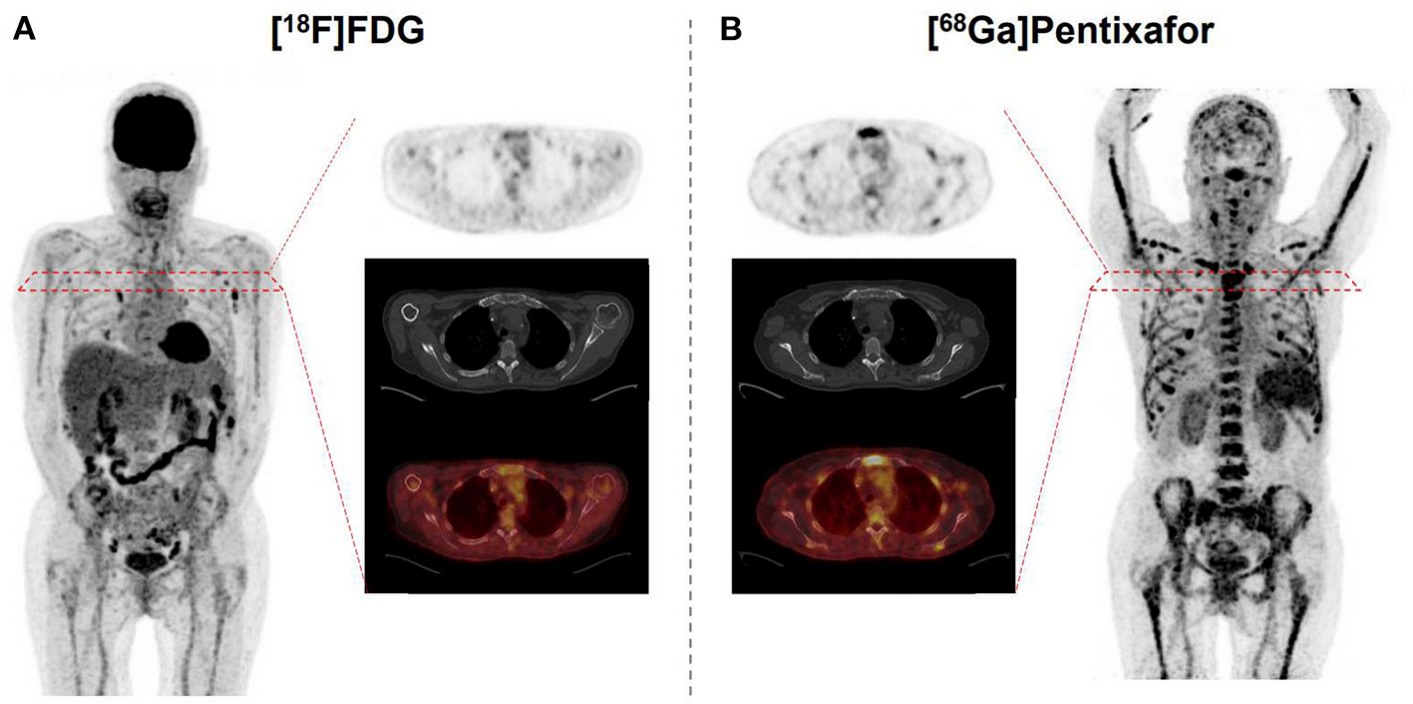
67 year-old patient afflicted with multiple myeloma. Relative to [^18^F]fluorodeoxyglucose (FDG) **(A)**, C-X-C motif chemokine receptor (CXCR4)-targeted [^68^Ga]Pentixafor **(B)** reveals more sites of disease, as displayed on both the maximum intensity projection and transaxial slices of PET (top) and PET/CT (bottom). Standardized uptake values range from 0 to 5 on both PETs.

In this regard, the ^68^Ga-labeled radiotracer PEN allows for assessing the expression levels of the receptor *in vivo* (31) and thus, has been investigated in various clinical settings (32). For instance, Lapa et al. evaluated the diagnostic potential of this novel radiotracer through a study of 35 heavily pretreated MM patients (33). In >65% of the cases, PEN identified CXCR4-expression at putative sites of disease, independent of clinical parameters such as MM subtype, cytogenetics, or serological findings. Its prognostic value was also evaluated, demonstrating that shortened time-to-progress and time-to-death were linked to CXCR4-positive lesions. In addition, a head-to-head comparison with FDG revealed an equal number of lesions in 42% of the cases, whereas in >20%, PEN was superior when compared to the reference radiotracer (33) (Figure 1).

Those retrospective results were further corroborated prospectively by Pan et al. recruiting 30 newly diagnosed MM patients who also underwent dual-radiotracer imaging with PEN and FDG (34). 28/30 (93.3%) patients had CXCR4-positive findings, while 16/30 (53.3%) patients had avidity on FDG PET, supporting the notion that PEN may identify additional sites of disease. Moreover, the authors reported on an association of PEN uptake with various clinical parameters, e.g., end organ damage or laboratory values reflecting tumor burden. For FDG, those correlations were less pronounced, further emphasizing the potential clinical value of PEN for a more precise reflection of disease extent (34).

Such promising results were recently expanded by Kuyumcu et al., who enrolled 24 MM patients to investigate the predictive performance of PEN (35). In line with existing literature, increasing PET-based CXCR4 positivity was linked to reduced overall survival, an observation that was more pronounced than the correlation of FDG with that parameter (35).

**Figure 2:**
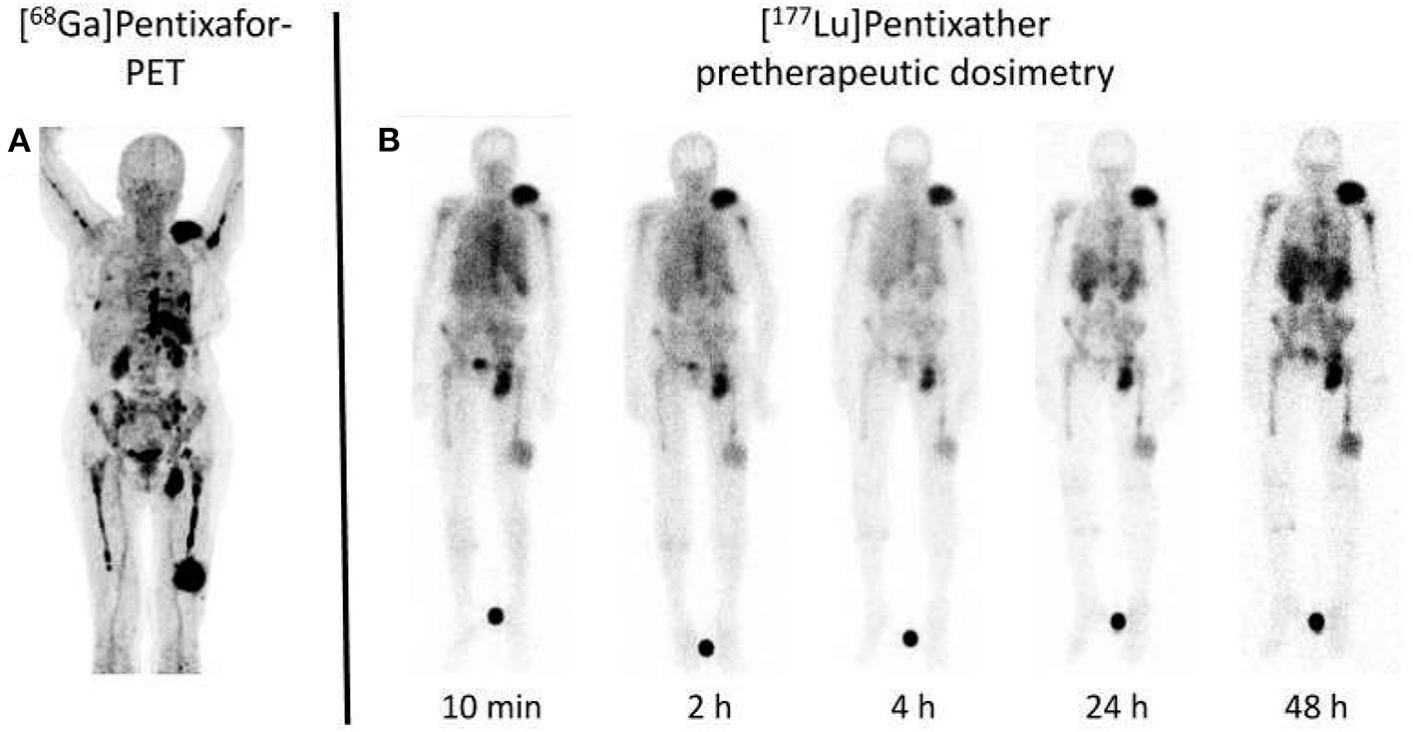
Dosimetry approach for C-X-C motif chemokine receptor (CXCR4) directed treatment. **(A)** Maximum intensity projection of [^68^Ga]Pentixafor PET in a multiple myeloma subject reveals multiple sites of both intra- and extramedullary disease involvement. **(B)** After injection of a small dosimetric activity of the therapeutic equivalent [^177^Lu]Pentixather (200 MBq), scintigraphy obtained at up to 48 h post-injection reveals long-lasting radiotracer accumulation at relevant sites of disease. Such an approach not only allows to assess myeloma doses in a therapeutic setting, but also provides an estimate of doses to the kidneys as organs of risk, thereby minimizing off-target effects. Modified from Lapa et al. (38).

Beyond its potential to overcome current limitations of FDG and its superior potential for risk stratification, PEN also allows the identification of patients eligible for treatment with CXCR4-targeted agents, the so called theranostic approach. As such, after CXCR4 expression in MM patients *in vivo* has been established by PET, the [^177^Lu]/[^90^Y]-labeled analog Pentixather can be administered intravenously for endoradiotherapy. Extensive preclinical and first-in-human studies (36), revealed remarkable response rates, even in advanced disease (37, 38), along with tolerable side effects (39). In those patients, [^177^Lu]Pentixather dosimetry was conducted prior to treatment onset to assess the appropriate amount of activity to be injected during therapy (Figure 2). As this therapeutic approach inevitably leads to bone marrow ablation, subsequent hematopoetic stem cell transplantation is necessary (37). The concept of PET-based imaging and therapy with PEN/[^177^Lu]Pentixather will also be further evaluated in the phase I/II COLPRIT trial (40).

### [^11^C]Methionine (MET)

**Figure 3:**
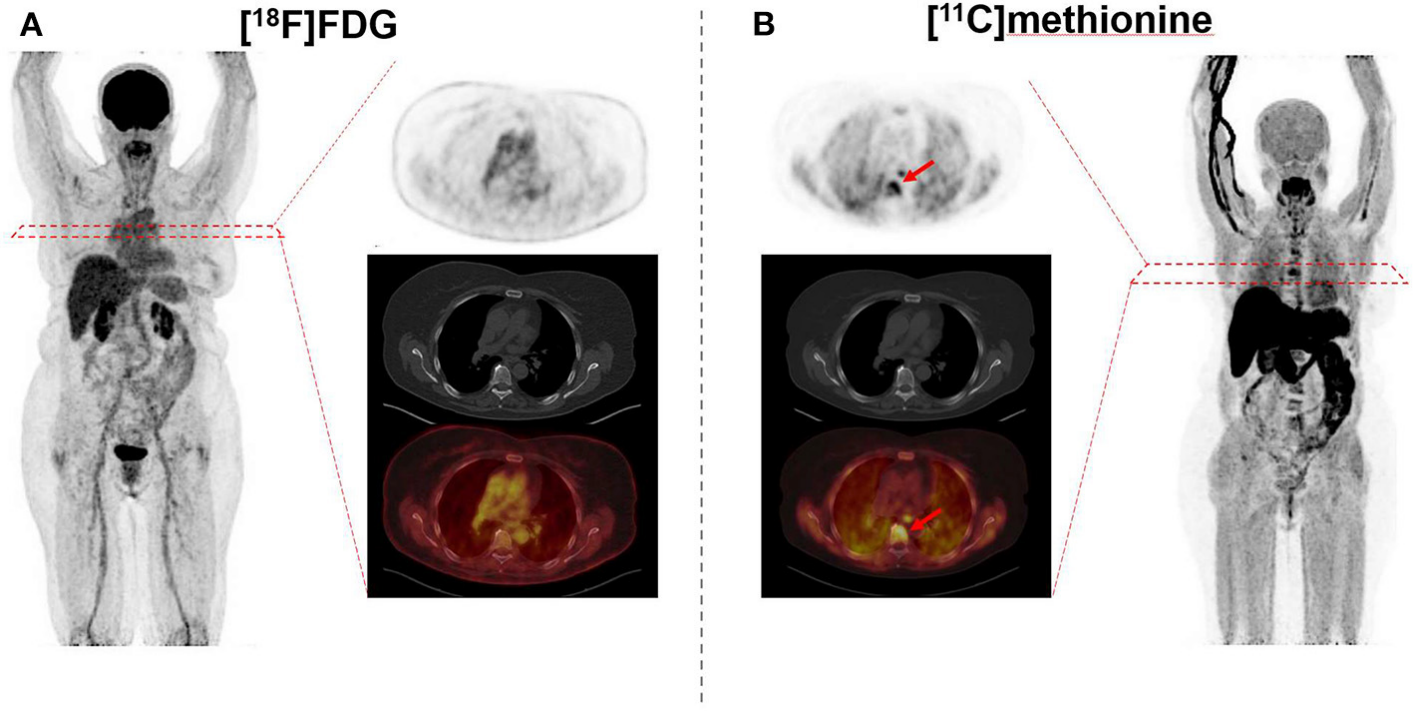
69 year-old female afflicted with multiple myeloma. Relative to [^18^F]fluorodeoxyglucose (FDG) **(A)**, [^11^C]methionine **(B)** reveals multiple intramedullary manifestations with intense uptake (arrows), as displayed on both the maximum intensity projection and transaxial slices of PET (top) and PET/CT (bottom). Standardized uptake values range from 0 to 5 on both PETs.

**Figure 4:**
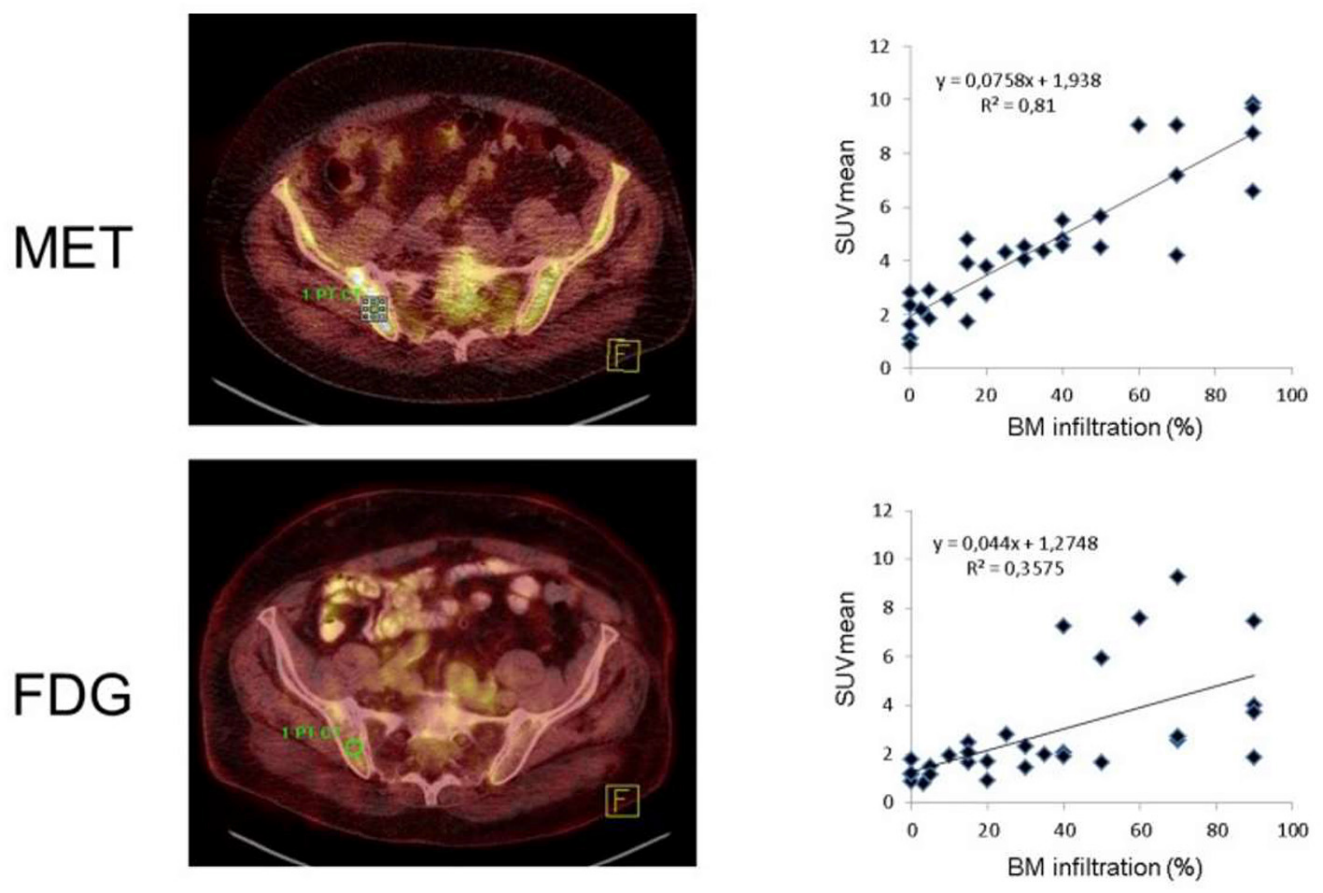
Radiotracer uptake reveals a tight link with bone marrow infiltration, which was more pronounced for [^11^C]methionine (MET, top) than for [^18^F]fluorodeoxyglucose (FDG, bottom). Bone marrow lesions identified by FDG and MET-PET (left). Right: Mean standardized uptake values (SUV_mean_) for all individuals [*n*(total) = 31] had a r of 0.6, while correlative index was even better for MET (r = 0.9). Modified from Lapa et al. (20).

Immunoglobulins produced by myeloma cells also offer a potential as a molecular imaging target. In this regard, rapid internalization of L-amino acids into MM cells for the synthesis of immunoglobulins suggested MET as an alternative radiotracer in MM patients (41–43). In a study of 19 subjects with MM, Nakamoto et al. performed a dual-radiotracer study by using MET and FDG prior to treatment and for restaging (44). MET had a higher accuracy of 93% when compared to FDG (86%), along with a greater number of MET-positive lesions, supporting the notion that MET may be superior for inconclusive findings obtained by FDG or for MM patients not adequately covered by conventional imaging (44). Further characterizing the clinical value of MET relative to FDG, Lapa et al. investigated newly diagnosed (11/43) and pretreated (32/43) MM patients by imaging with both FDG and MET (20) (Figures 3, 4). MET PET detected MM lesions in 39/43 (90.7%) patients, whereas PET with FDG was considered positive only in 33/43 (76.7%) of the subjects. In addition, MET PET revealed more focal intra- and extra-medullary lesions in 28/43 (65.1%) patients. The extent of bone marrow infiltration, which was confirmed histologically in 32 patients, positively correlated with both MET and FDG uptake, which was more pronounced for the amino acid radiotracer (20).

Those promising results were further corroborated in a follow-up study that pooled 78 patients with MM from two different centers in Germany and Spain. A direct FDG/MET comparison revealed that active sites of disease would have been missed in 12/78 (15.3%) patients if only FDG would have been used (45). To provide evidence on the superior specificity of MET relative to FDG, biopsy was conducted in two patients, and the histology was concordant with MET findings [FDG(+)/MET(−) demonstrating no plasma cell infiltration vs. Case #2, FDG(−)/MET(+) having infiltration of clonal plasma cells] (45). As such, MET may replace FDG as current reference for molecular imaging in MM patients (45). PET-based semiquantitative parameters were also assessed and, relative to FDG, MET-derived image biomarkers demonstrated a more substantial correlation with tumor burden, suggesting MET-based PET quantification as a novel tool to identify high-risk individuals (46).

### [^11^C]Choline (CH)

Choline has several physiological functions, including participation in cell growth and in the cell membrane synthesis as a component of phosphatidylcholine. The retention of choline is attributed to several uptake mechanisms. In cancer cells, including MM cells, uptake may be significantly increased compared to normal tissue due to the high replication rate and associated increased cell membrane turnover (47, 48). With approval of CH in individuals with prostate cancer, this tracer may be used more widely in the United States and Europe for investigating MM (49). Due to the 20 min half-life of [^11^C], CH is limited to PET centers with access to an on-site cyclotron. Alternatively, CH can also be linked to [^18^F], providing a half-life of 110 min. However, robust data of a direct comparison between both radiotracers is still missing. In this regard, results of a currently recruiting trial investigating the performance of FDG relative to [^18^F]-choline are awaited (50). As results on [^18^F]-choline in MM are rather limited, we will focus on CH.

Nanni et al. were the first conducting a comparison between CH and FDG in 10 patients with advanced MM at different disease stages, with a higher rate of bone lesions detected by CH relative to the reference radiotracer FDG (17). Based on these encouraging findings, Lapa et al. compared MET and CH in 19 subjects with MM (18/19) or solitary bone plasmacytoma (1/19) (22). The authors demonstrated that MET was more accurate than CH to detect active MM lesions, along with an improved target-to-background ratio, and a substantial association between bone marrow involvement and MET accumulation (22).

Nonetheless, CH PET like MET PET is currently not applied to MM on a regular basis and may be further included in daily routine after having proven better accuracy in larger clinical trials. In this regard, 30 newly diagnosed MM patients will be evaluated by [^18^F]fluorocholine and FDG in a currently recruiting study (51). This investigations aims to segregate between true- vs. false-positive PET lesions by comparing those scans with whole-body MRI (51), with the latter imaging modality known to be the reference for bone marrow lesions (52).

### [^18^F]-3′-Fluoro-3′-Deoxythymidine (FLT)

Interacting with cytosolic thymidine kinase (TK1) of the cell cycle, FLT represents a read-out of proliferating cells (53). The number of studies investigating FLT in the context of MM are limited. For instance, Sachpekidis et al. investigated FDG and FLT in eight MM subjects and found a total of 48 FDG positive lesions, while FLT identified 17 lesions. The latter radiotracer was also challenging for the assessment of lesions allocated to the bone marrow, mainly due to increased background (54).

### [^18^F]-Sodium Fluoride (NaF)

NaF is widely utilized for assessment of bone involvement (55, 56). Not surprisingly, this radiotracer has also been applied to MM. Including 60 patients, FDG was compared to NaF and the latter radiotracer identified significantly less lesions attributable to MM (FDG, 343 vs. NaF, 135) (57). Those findings were further corroborated in a recent study assessing treatment response in MM patients after chemotherapy and stem cell transplantation. Of 129 FDG(+) lesions, the bone-seeking radiopharmaceutical identified approximately half sites of disease. Taken together, the authors concluded on a limited role of NaF for assessing therapeutic efficacy (58).

## Future Perspectives

Given already favorable results of PEN imaging in patients with MM (38), the recent introduction of novel, second-generation CXCR4-directed radiotracers may improve efficacy along with prolonged tumor retention, thereby allowing for better image contrast, increased lesion detection rate, and even improved outcome (59) for therapeutic agents.

In addition, direct comparison of CXCR4-targeted radiotracers to MET, e.g., to reveal complementary information or further insights into complex tumor heterogeneity in advanced disease, should be a goal of the field moving forward. This has already been conducted in smoldering MM by comparing FDG, MET and (first-generation radiotracer) PEN, with the latter agents MET and PEN being more accurate in detecting bone marrow involvement. Given these encouraging results, such triple-tracer approaches could be expanded toward other clinical scenarios, e.g., to identify high-risk smoldering MM patients eligible for clinical trials (60).

The high sensitivity of MET might prove especially clinically useful for residual disease, while FDG has demonstrated substantial value in addition to bone marrow based approaches (6).

In recent years, hybrid devices including PET and magnetic resonance imaging (MRI) have been installed. In cancer patients, Beiderwellen et al. were among the first to report on an improved read-out of bone lesions using PET/MRI when compared to PET/CT (61). Based on these encouraging results, 30 MM patients were investigated using PET/MRI and PET/CT. After FDG injection, there were no relevant differences on a visual and quantitative assessment between both imaging modalities, with more than 94% of sites of disease identified both on PET/CT and PET/MRI. Future studies may further determine the usefulness of PET/MRI in MM patients, e.g., to assess treatment response (62).

Last, future studies should also investigate the potential of machine learning in the context of molecular imaging for MM patients, potentially increasing diagnostic accuracy (63). Such an approach may also allow for better identification of individuals who might benefit from a CXCR4-targeted radiolabeled therapeutic intervention or could allow the identification of high-risk individuals prone to off-target effects, e.g., in the kidneys (39).

## Conclusion

Considered the work-horse for molecular imaging in MM patients, FDG is endorsed by current guidelines for clinical situations such as response monitoring. The inherent limitations of this radiotracer have triggered the development of (novel) alternative PET radiopharmaceuticals. Among others, the CXCR4-targeted agent PEN, the amino acid radiotracer MET and CH have been extensively investigated, demonstrating increased sensitivity and specificity when compared to FDG. Of note, PEN also paves the way to select candidates for a theranostic approach, offering a rationale for CXCR4-based endoradiotherapy in advanced disease. Further studies are needed, e.g., evaluating PEN and MET in a prospective setting and to prove their clinical impact on therapeutic decisions, as well as to establish improved algorithms to identify subjects who might benefit from CXCR4-targeted radiolabeled therapy by minimizing off-target effects.

## Author Contributions

All authors contributed in drafting, revising, and proofreading the manuscript.

## Conflict of Interest

The authors declare that the research was conducted in the absence of any commercial or financial relationships that could be construed as a potential conflict of interest.

## Publisher's Note

All claims expressed in this article are solely those of the authors and do not necessarily represent those of their affiliated organizations, or those of the publisher, the editors and the reviewers. Any product that may be evaluated in this article, or claim that may be made by its manufacturer, is not guaranteed or endorsed by the publisher.
